# Response to “Improvement of female pattern hair loss with bicalutamide: A time-dependent process”

**DOI:** 10.1016/j.jdin.2025.08.006

**Published:** 2025-08-29

**Authors:** Ricardo da Silva Libório, Hélio Amante Miot, Paulo Müller Ramos

**Affiliations:** Departamento de Dermatologia, FMB-UNESP, Botucatu, São Paulo, Brazil

**Keywords:** androgenetic alopecia, bicalutamide, female pattern hair loss, oral minoxidil, treatment, woman

*To the Editor:* we appreciate the interest of Gil-Redondo et al in our study and agree that the antiandrogenic effect of bicalutamide may require a longer duration to become clinically apparent in female pattern hair loss (FPHL). Retrospective studies have reported that 17% to 28% of patients show clinical improvement within the first 6 months of treatment^1^^,^^2^ however, such early improvement was not observed in our randomized clinical trial.^3^

It is important to highlight that our trial was the first to assess bicalutamide in combination with oral minoxidil—reflecting common clinical practice—rather than as monotherapy. This design complicates direct comparison with studies evaluating antiandrogens alone. When 2 effective agents are combined, the incremental benefit of 1 may be less apparent, particularly if 1, such as oral minoxidil, exerts a faster and more robust effect on hair density. In such cases, the impact of bicalutamide could be masked during a relatively short follow-up period.

Moreover, although antiandrogens are widely prescribed in FPHL, the overall level of evidence supporting their efficacy remains low, and the role of androgenic mechanisms in FPHL pathophysiology is still debated.^4^^,^^5^ Most patients with FPHL do not present with a hyperandrogenic phenotype (eg, hirsutism, acne, seborrhea), and stratification by phenotype may reveal differential responses to antiandrogen therapy.

Our findings suggest that bicalutamide 25 mg/d may be less rapid or potent than previously inferred from retrospective reports. Higher doses (eg, 50 mg/d) might produce more robust effects, but dose-ranging randomized trials are warranted.

The mechanisms underlying hirsutism differ from those of facial hypertrichosis induced by minoxidil. The way bicalutamide mitigates facial hypertrichosis and hair shedding remains uncertain; nonetheless, it may represent a strategy to allow higher doses of minoxidil while limiting these adverse effects.

Finally, we agree that larger and longer randomized trials are needed to fully define the role of bicalutamide—and antiandrogens in general—in FPHL management. Nevertheless, our trial contributes to the literature by suggesting that early effects may be more modest than those reported in retrospective studies.

## Conflicts of interest

None disclosed.
